# Correlations between subfoveal choroidal thickness, macular thickness, and visual outcome in neovascular age-related macular degeneration using swept source OCT: insights from intravitreal aflibercept treatment

**DOI:** 10.1186/s40942-023-00506-4

**Published:** 2023-11-15

**Authors:** Daniel P. Beraldo, Marcussi P. Rezende, João G. Alexander, Júlia Polido, Rubens Belfort, Thiago Cabral

**Affiliations:** 1 Clínica Oftalmo-Retina, Presidente Prudente, SP Brazil; 2 Department of Ophthalmology, Federal University of São Paulo (UNIFESP), São Paulo, SP 04039-032 Brazil; 3 Department of Specialized Medicine, CCS and Vision Center Unit, Ophthalmology, EBSERH/HUCAM, CCS-UFES-Federal University of Espírito Santo (UFES), Vitória, ES 29047-105 Brazil

**Keywords:** Age-related macular degeneration, Neovascular, Aflibercept, Macular thickness, Choroidal thickness, OCT Swept Source

## Abstract

**Background:**

Age-related macular degeneration (AMD) is a leading cause of visual impairment among individuals aged 50 and above, often resulting in irreversible vision loss (1). Currently, antiangiogenic therapy is the primary treatment approach for neovascular AMD (2). The choroid has gained significant attention in recent years due to its involvement in various ocular pathologies (7). The objective of this study was to evaluate visual acuity and correlate pre-treatment variables, such as foveal thickness and choroidal thickness, with post-treatment outcomes.

**Materials and Methods:**

This study was designed as a prospective interventional study to investigate the changes in choroidal and macular thickness in patients with neovascular AMD who received intravitreal aflibercept injections. The study utilized medical records and employed Swept Source Optical Coherence Tomography (OCT-SS) for evaluation. The data was collected from patients treated in Presidente Prudente, Brazil, during a three-month load dose period.

**Results:**

The best-corrected mean visual acuity significantly improved from 1.0 logarithm of the minimum resolution angle (logMAR) units to 0.55 logMAR after treatment with aflibercept (p < 0.001). Patients undergoing treatment exhibited a significant decrease in average macular thickness from 323 μm to 232 μm (p = 0.001), as well as a reduction in choroidal thickness from 206 μm to 172 μm (p = 0.031), while maintaining intraocular pressure within the normal range (p = 0.719) without significant variation. Statistically significant associations were found between the difference in pre- and post-treatment choroidal thickness and the pretreatment values of macular thickness (p = 0.005) and choroidal thickness (p = 0.013). There was also a statistically significant correlation between the difference in pre- and post-treatment macular thickness and the pretreatment macular thickness value (p < 0.001).

**Conclusion:**

In this study, aflibercept exhibited remarkable effectiveness in reducing macular and choroidal thickness, as evaluated using OCT-SS, and significantly improved visual acuity in patients with neovascular AMD. The assessment of both choroidal and macular changes, as well as their correlations, can provide valuable insights for clinicians, enabling them to make well-informed therapeutic decisions and effectively monitor treatment outcomes. Notably, this study contributes to the existing body of literature as the first to establish a correlation between pretreatment foveal thickness, variation in choroidal thickness, and post-treatment choroidal thickness.

## Introduction

Age-related macular degeneration (AMD) is a leading cause of visual impairment in individuals over 50 years of age in Western countries and can result in irreversible vision loss [1, 2]. Due to population aging, there is a predicted increase in AMD cases in the USA from 2.7 million in 1970 to 7.5 million in 2030 [2]. In Brazil, there is no population-based study assessing the real impact of AMD, but it is estimated that 3 million elderly people have AMD at different stages [3]. Other risk factors for AMD, aside from age and white race, include female gender, arterial hypertension, hypercholesterolemia, cardiovascular disease, obesity, positive family history, smoking, high levels of C-reactive protein and other inflammatory markers, hyperopia, and light-colored iris. Smoking has been consistently shown to be the most significant modifiable risk factor [1].

AMD can be classified into dry or neovascular types. Neovascular AMD is characterized by choroidal neovascularization (CNV), which is the growth of choroidal neovessels in spaces between the retinal pigmented epithelium (RPE) and Bruch's membrane and/or between the sensory retina and RPE. Although less prevalent, accounting for 10% of total cases, neovascular AMD is responsible for approximately 80% of total legal blindness cases attributed to AMD [4–8].

Several biomarkers have been detected in AMD, such as druse characteristics, geographical atrophy, subretinal fluid, and detachment of the RPE [9]. The introduction of enhanced depth image optical coherence tomography (OCT-SS) has provided better penetration of choroidal signal using increased wavelengths [10–12]. In addition to the evidence suggesting the dynamic role of choroidal circulation in the pathogenesis of AMD, the optical coherence tomography (OCT) device provides additional information on choroidal thickness in AMD. It has been shown that patients with AMD have thinner choroidal thickness than normal controls matched by age [13].

Although the impact of choroidal circulation and its thickness has been implicated in the pathogenesis of AMD, the relationship between choroid and macular thickness to visual outcome and response to treatment after therapy with anti-vascular endothelial growth factor (VEGF) has not been well documented in patients with neovascular AMD [7]. In this study, for the first time in literature, we assessed the subfoveal choroidal thickness and macular thickness of the eyes, using OCT-SS, with neovascular AMD and its association with response to treatment after intravitreal injections of aflibercept during the 3-month load dose follow-up. In addition, we investigated the prognostic implication of subfoveal choroidal thickness, macular thickness, and the relation between them in response to treatment and visual outcome in patients with neovascular AMD.

## Methods

A prospective interventional study was conducted as a case series, involving a sample of 15 patients diagnosed with neovascular AMD who had not received prior treatment (naive) and were recommended for intravitreal injections of aflibercept. The study was conducted, and the patients who met the inclusion criteria received treatment at Clínica Oftalmo-Retina in Presidente Prudente, SP.

### Inclusion criteria

(a) Patients with neovascular monocular AMD, with type 2 neovascular membrane (subretinal), who were treatment-naive and received three intravitreal injections of aflibercept during the specified period, with the same ophthalmologist administering the injections. (b) Complete medical records with research data, including sex, age, duration of illness, current medications, pre and postoperative intraocular pressure (IOP), corrected visual acuity (VA) before and after the procedure, and OCT-SS scans. Patients should not have received any previous intravitreal anti-VEGF treatments. (c) Patients who provided signed Free and Informed Consent Forms, which were filled with their medical records.

### Exclusion criteria

(a) Patients who received previous treatments for neovascular AMD, such as photodynamic therapy or intravitreal injections. (b) Patients who did not complete the proposed treatment of three intravitreal injections. (c) Patients who did not undergo OCT scans after the completion of three intravitreal injections until the date of the research. (d) Patients who underwent previous glaucoma surgery. (e) Patients who discontinued the treatment. (f) Patients who did not provide a signed Informed Consent Form. (g) Patients with neovascular binocular AMD.

### Ethical Committee

The study was conducted in compliance with the guidelines outlined in Resolution 196/96 of the National Health Council of the Ministry of Health. The research protocol was submitted for analysis and received approval from the Ethics Committee of the Hospital Regional do Câncer da Santa Casa de Misericórdia de Presidente Prudente – SP (CAAE—19386619.1.0000.8247).

### Execution

All individuals who met the inclusion criteria and provided written informed consent between April 2018 and January 2020 were recruited for the study. After the diagnosis of neovascular AMD and data collection, the patients were evaluated based on the following criteria:

(a) Sex and age (b) Duration of illness (c) Best-corrected visual acuity (VA) (d) Pre- and postoperative intraocular pressure (IOP) analysis e) Analysis of OCT-SS scans before and after the procedure to determine variations in central macular thickness and choroidal thickness.

Ophthalmological examinations were conducted on patients during their follow-up visits, which included taking their medical history, assessing VA with better correction, examining the anterior segment using a slit lamp (biomicroscopy), performing Goldmann applanation tonometry, conducting posterior segment ophthalmoscopy (retinal mapping), angiography, and OCT-SS imaging using the Triton Plus 3D Range Imaging device from Topcon Corporations, Tokyo, Japan. The specific measurement parameters employed in OCT-SS to assess neovascular membrane activity include the presence of subretinal and/or intraretinal fluid (cysts), fusiform thickening in the RPE-choriocapillaris complex, separation of the retinal pigment epithelium (indicating the presence of fluid or bleeding in this region), and subretinal hemorrhage.

Once neovascular AMD was diagnosed and treatment with intravitreal aflibercept injection was indicated, patients were provided with information about the procedure, its risks, benefits, and visual prognosis. They were given a Free and Informed Consent Form to read and sign, and a copy was filed in their medical records.

The intravitreal injection was performed in a surgical center by the same experienced ophthalmologist following a specific protocol: PRN (pro re nata)—the frequency was one application every 4 weeks, until completing 3 intravitreal injections (loading dose). After the 3 initial applications, all patients continued to be evaluated at the outpatient clinic, and those who continued to show signs of neovascular membrane activity continued to be treated.

The procedure involved applying anesthetic eye drops and 5% povidone iodine to the conjunctival sac, performing asepsis using aqueous chlorhexidine on the eyelashes, eyelids, and periorbital skin, placing a fenestrated field and blepharostat, and then injecting intravitreal aflibercept 0.05 ml (2 mg solution with a concentration of 40 mg/ml) via pars plana using a 0.3 × 13 mm needle, 30 Gauge, positioned 3.5 mm from the limbus. Pressure was applied to the injection site using a sterile cotton swab for one minute to prevent leakage. Patients were instructed to apply antibiotic eye drops (0.3% tobramycin) postoperatively every 6 h for 7 days.

Patients were followed up on an outpatient basis on the first and twenty-first postoperative days, and the next intravitreal injection was scheduled with a 4 week interval between injections. After the completion of the three intravitreal applications, they were evaluated for the need for additional injections based on the OCT-SS examination.

**Fig. 1:**
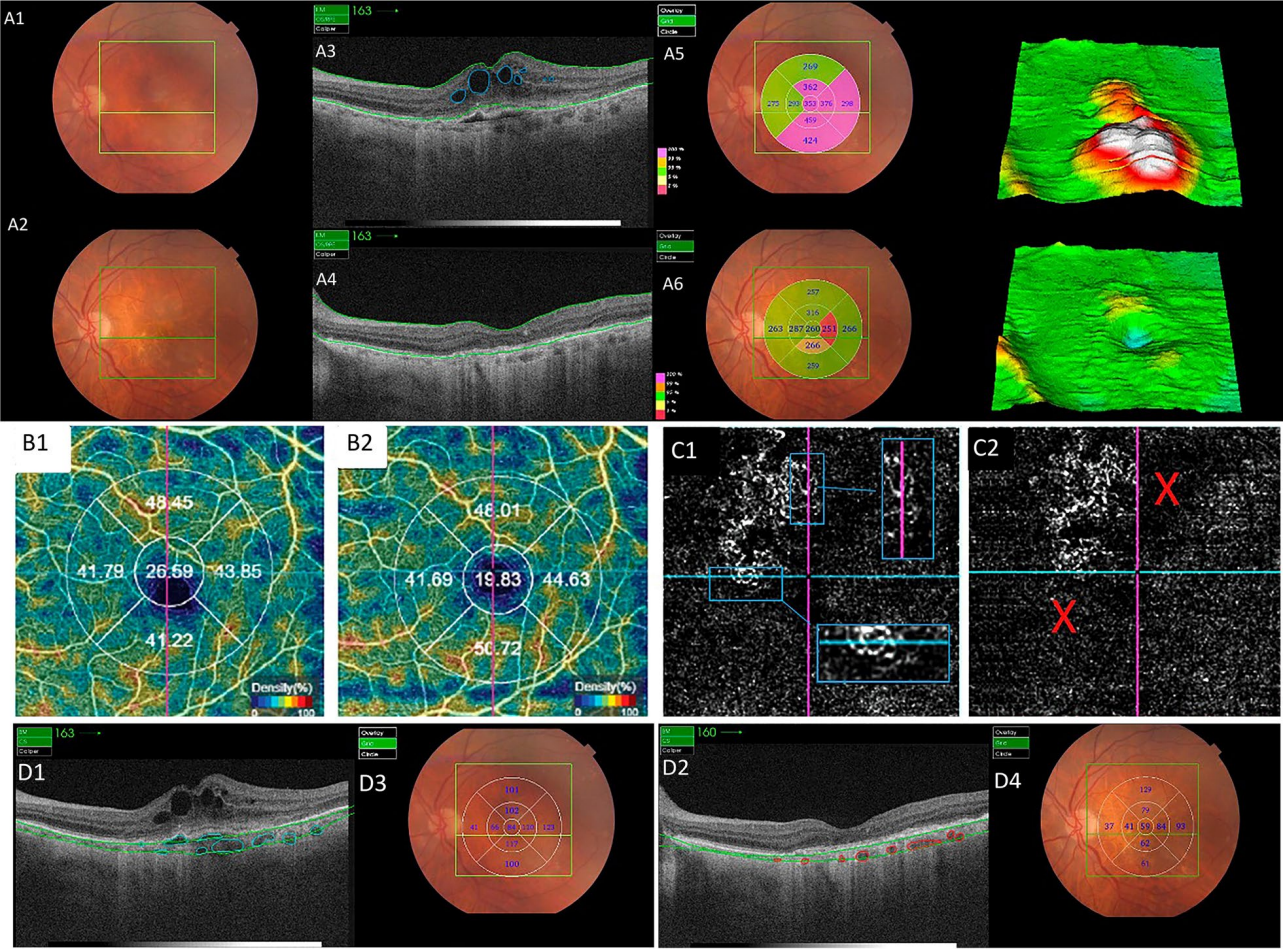
Multimodal images of a patient included in the study, depicting neovascular age-related macular degeneration treated with three aflibercept injections. **A** Retinography (A1: pre-treatment and A2: post-treatment). OCT B-scan: Pre (A3) and post-treatment (A4) images demonstrate macular thickness reduction after treatment. Figures A5 and A6 display retinography with the central foveolar thickness measurement map before and after treatment: 353 and 256 microns, respectively. **B** OCT-Angiography Density map: pre (B1) and post-treatment (B2). **C** OCT-angiography (outer retina) figures: C1 and C2 illustrate the neovascular membrane before and after treatment. **D** Pre (D1) and post-treatment (D2) OCT B-scan images showing choroidal thickness, also displaying a reduction after treatment. Figures D3 and D4 depict retinography with the central choroidal thickness measurement map before and after treatment: 84 and 58 microns, respectively

Choroidal thickness was measured using the digital calipers tool available on the OCT-SS device (Triton Plus 3D Range Imaging; Topcon Corporations, Tokyo, Japan) in 15 eyes of 15 patients with neovascular AMD before and after treatment with anti-VEGF (aflibercept). An independent researcher, who was not involved in performing the injections and was masked to the clinical data of each patient, performed the measurements. Automatic choroidal design was initially conducted, followed by manual adjustment if the researcher observed that the automatically registered area was not properly centered on the fovea. Finally, an analysis of macular thickness was performed, and statistical analysis was conducted (Fig. 1).

The OCT-SS images, as well as the injections, were obtained by the same experienced ophthalmologist both before the treatment and after the three-monthly applications of intravitreal aflibercept. The scanning acquisition was performed in low ambient light conditions, with the patient fixating on an internal fixation point to achieve the best alignment when fixation was present.

### Data analysis

To evaluate the distribution of the analyzed groups, a Gaussian distribution test was conducted. Mean and standard deviation analyses were performed, and for groups that demonstrated a normal distribution, the student-t test was employed to compare the pre and post groups. In cases where a normal distribution was not observed, the Wilcoxon test was utilized for the pre and post comparisons.

Correlation analyses between the results were performed using Pearson’s correlation coefficient for groups with a normal distribution, while Spearman's correlation coefficient was employed for groups without a normal distribution. The thickness of the choroid was categorized into four groups based on static analysis, resulting in the following categories: (a) 70–139 μm, (b) 140–209 μm, (c) 210–279 μm, and (d) 280–349 μm. Correlations between choroidal thickness and other variables, such as visual acuity and foveal thickness, were then examined.

To quantify the reduction in macular and choroidal thickness, the relative delta, expressed as a percentage, was calculated using the formula: (initial value—final value)/initial value × 100. A significance level of 5% was adopted for all statistical analyses.

## Results

This study enrolled 15 treatment-naive patients with neovascular age-related macular degeneration (AMD), with one eye per patient. The patients received three injections of aflibercept, with a 28 day interval between each application. Various parameters were assessed, including gender, age, change in intraocular pressure (IOP) after the procedure, change in corrected visual acuity (VA), and variations in macular and choroidal thickness measured by OCT-SS (Tables 1, 2, 3, 4, 5). All 15 patients were treatment naive, and no complications related to the procedure were observed.

**Table 1:** Description of patients' personal and clinical characteristics

Characteristic	
*N (pacients)*	15
*Age*	79.80 ± 7.34 (81)
*Gender*	
Female	5 (33.33%)
Male	10 (66.67%)
*Eye*	
Right	8 (53.33%)
Left	7 (46.67%)
*Personal background*	
Cardiopathy	2 (13.33%)
DM II	3 (20.00%)
SAH	9 (60.00%)
Hypothyroidism	1 (6.67%)
Parkinson	1 (6.67%)
No comorbidities	3 (20.00%)
*Phakic/Pseudophakic*	
Phakic	2 (13.33%)
Pseudophakic	13 (86.67%)

**Table 2:** Frequency of preoperative OCT-SS findings

Variable	N (%)
Intraretinal fluid	
Yes	9 (60.00)
No	6 (40.00)
Subretinal fluid	
Yes	12 (80.00)
No	3 (20.00)
RPE detachment	
Yes	7 (46.67)
No	8 (53.33)

**Table 3:** Description of ocular parameters at each moment of evaluation and result of comparative tests

Characteristic	Pre	Post	Diference	P-value^a^
VA (logMAR)	1.00 ± 0.39 (1.00)	0.55 ± 0.27 (0.48)	− 0.45 ± 0.22 (− 0.40)	0.0006
Macular thickness (μm)	323.40 ± 103.39 (284)	231.60 ± 64.48 (227)	− 91.80 ± 89.46 (− 83)	0.0010
Subfoveal choroidal thickness (μm)	206.33 ± 92.96 (226)	172.00 ± 87.16 (172)	− 34.33 ± 55.45 (− 17)	0.0054
IOP (mmHg)	12.27 ± 0.88 (12)	12.20 ± 0.77 (12)	− 0.07 ± 0.70 (0)	0.9411

^a^P-value for the Wilcoxon signed-rank test. Values less than 0.05 show a statistically significant difference between groups

**Table 4:** Average reduction in subfoveal choroidal thickness in relation to basal thickness itself

Subfoveal choroidal thickness (μm)	_n_	Average reduction (μm)	P-value
70–139	4	18.3 ± 6.1	0.009
140–209	3	9.0 ± 15.0	0.408
210–279	4	44.3 ± 32.9	0.075
280–349	3	59.5 ± 105.4	0.341

**Table 5:** Variation of VA in logMAR after treatment in relation to basal subfoveal choroidal thickness

Subfoveal choroidal thickness (μm)	n	VA average reduction (logMAR)	P-value
70–139	4	0.35 ± 0.2	0.044
140–209	3	0.70 ± 0.3	0.056
210–279	4	0.40 ± 0.2	0.011
280–349	3	0.41 ± 0.2	0.019

Significant reductions were observed in both macular and choroidal thickness after the three injections–Fig. 1. The average macular thickness decreased from 323.40 μm to 231.60 μm, while the choroidal thickness reduced from 206.33 μm to 172.00 μm. The best-corrected mean VA also improved significantly from 1.0 logarithm of the minimum resolution angle (logMAR) units to 0.55 logMAR after treatment with aflibercept (p < 0.001). The IOP remained within the normal range after the injections, with only minor fluctuations that can be attributed to diurnal variations (Table 3).

Correlation analyses were conducted to examine the relationships between different OCT-SS parameters (Table 6). It was observed that higher pretreatment macular thickness was associated with smaller pre/post differences in both macular thickness (p = 0.0051) and choroidal thickness (p = 0.03). Furthermore, greater pretreatment choroidal thickness was linked to a larger increase in post-treatment choroidal thickness (p = 0.0009). The study also revealed that larger differences in macular thickness between pre and post-treatment were associated with greater differences in choroidal thickness (p = 0.0218).

**Table 6:** Correlations between the different OCT-SS parameters, incluiding macular and choroidal thickness pre/post treatment and the difference between them

	Macular thickness pre	Macular thickness post	Macular thickness difference	Choroidal thickness pre	Choroidal thickness post	Choroidal thickness difference
Macular thickness pre						
Macular thickness post	0.3861 0.1552					
Macular thickness difference	− 0.6821 **0.0051**^a^	0.2842 0.3046				
Choroidal thickness pre	− 0.2786 0.3147	− 0.1626 0.5625	0.1250 0.6571			
Choroidal thickness post	− 0.6357 **0.0109**^a^	− 0.3771 0.1658	0.3929 0.1475	0.7643 **0.0009**^a^		
Choroidal thickness difference	− 0.5464 **0.0351**^a^	− 0.0286 0.9194	0.5857 **0.0218**^a^	− 0.2143 0.4431	0.2429 0.3831	

^a^In bold, correlations with statistical significance (p < 0.05) are highlighted

Statistically significant associations were found between the pre/post difference in choroidal thickness and the pretreatment values of macular and choroidal thickness (Table 7). Similarly, Table 8 demonstrates a significant association between the pre/post difference in macular thickness and the pretreatment macular thickness value.

**Table 7:** Result of multiple linear regression to identify factors associated with the pre/post difference in choroidal thickness

	Coefficient (95% CI)	p-value
Pre macular thickness	− 0.31 (− 0.54 a − 0.09)	0.005
Pre choroidal thickness	− 0.31 (− 0.56 a −0.07)	0.013

**Table 8:** Multiple linear regression for pre/post macula thickness difference

	Coefficient (95% CI)	p-value
Pre macular thickness	− 0.68 (− 0.99 a − 0.37)	< 0.001
Pre choroidal thickness	0.01 (− 0.34 a 0.35)	0.959

**Fig. 2:**
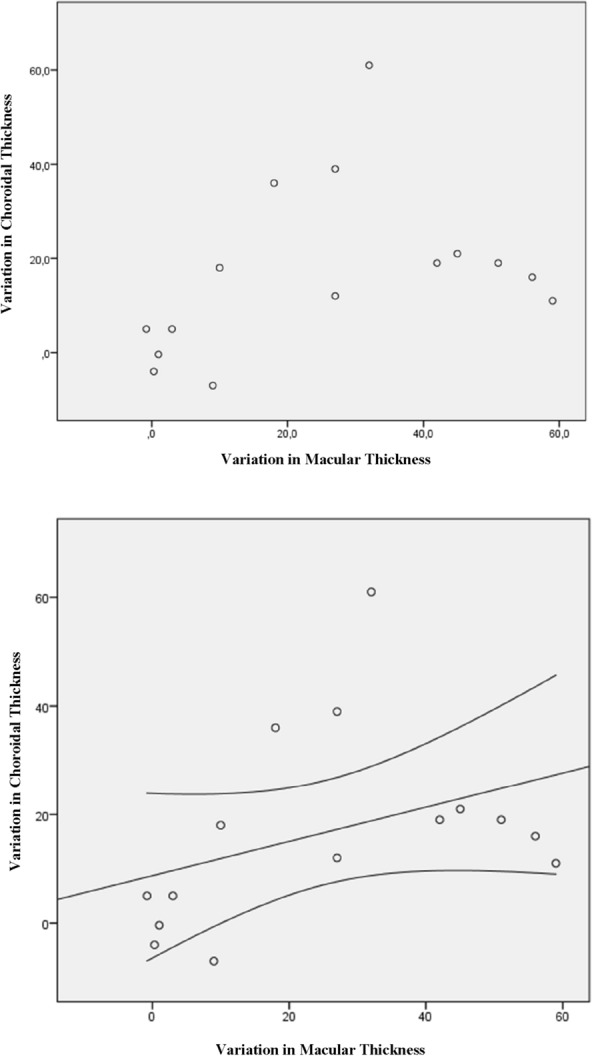
Variation in choroidal thickness vs variation in macular thickness (p = 0.165)

**Fig. 3:**
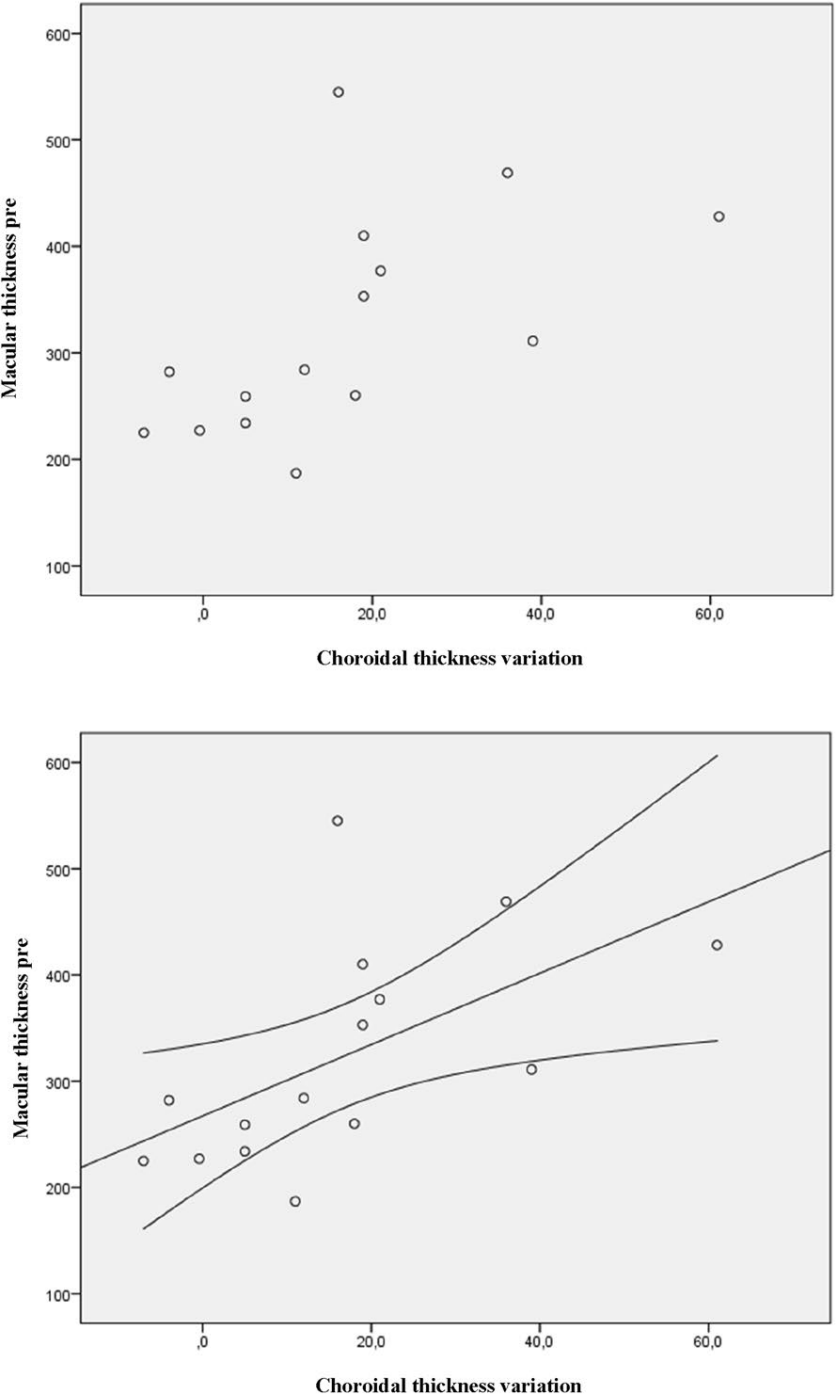
Macular thickness pre VS choroidal variation (p = 0.023)

**Fig. 4:**
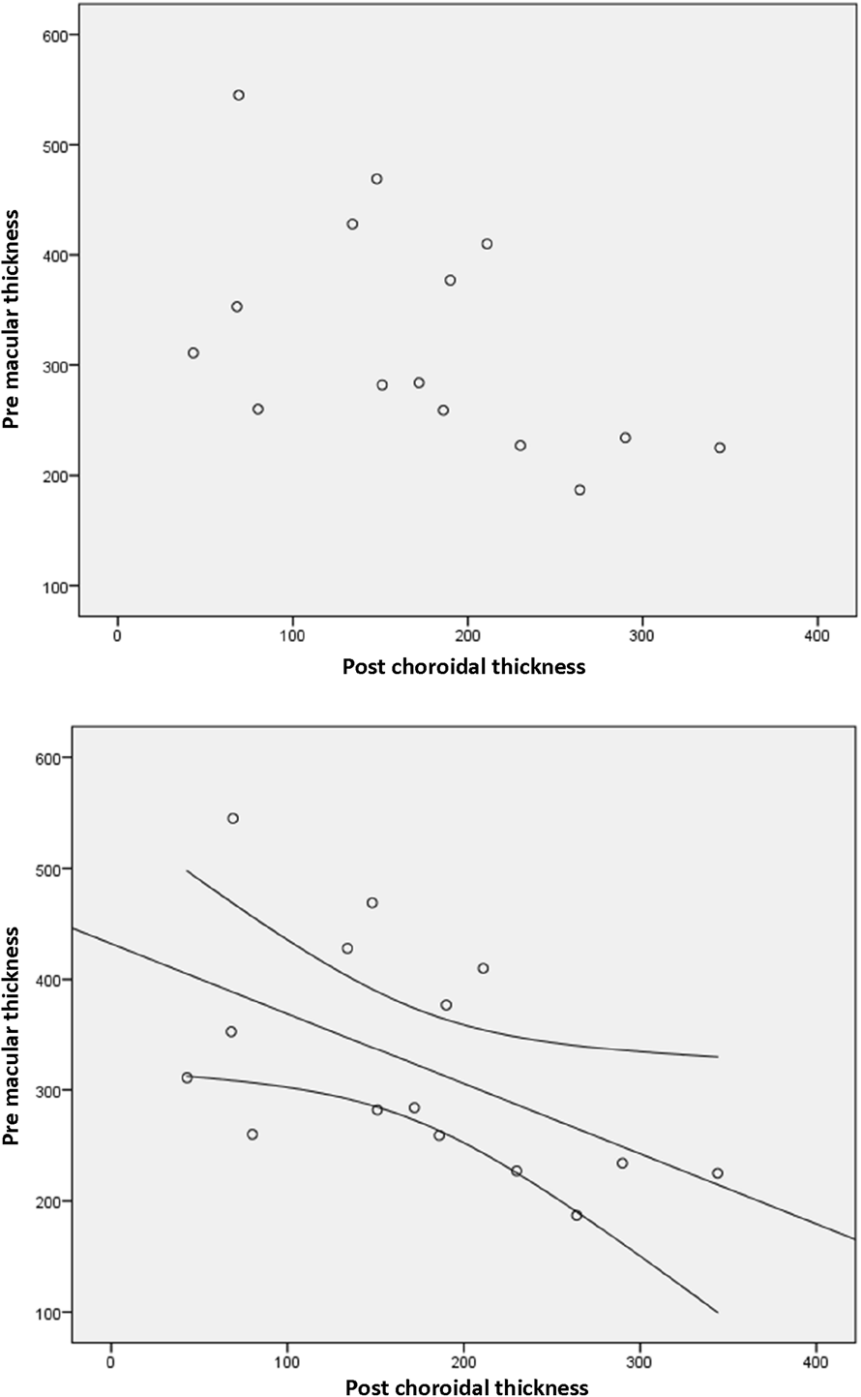
Pre macular values correlated with Post choroidal values (p = 0.041)

Figures 2, 3, and 4 illustrate the statistically significant correlations between choroidal thickness and variation in macular thickness, macular thickness pretreatment, and choroidal thickness variation. Additionally, they depict the association between pre macular values and post choroidal values. Figure 2 shows: Choroidal thickness variation versus macular thickness variation (p = 0.165). The trend line demonstrates a weak and positive correlation, that is, the greater the choroidal reduction value, the greater the macular reduction value. There was no statistical significance in this analysis. Figure 3 shows: Pre macular thickness X choroidal variation (p = 0.023). The correlation seen above is moderate and positive, with the trend line highlighting that the greater the pre macular thickness, the greater the decrease in choroidal thickness, which is statistically significant. And Fig. 4 shows us the RPE macular values correlated with post choroidal values (p = 0.041). Finally, the trend line with a moderate and negative correlation, that is, the higher the pre macular value, the lower the post choroidal value, with statistical significance.

## Discussion

Visual outcomes and treatment response after three monthly intravitreal injections of aflibercept were significantly correlated with subfoveal choroidal thickness. In healthy individuals, the average choroidal thickness in the central macular region is approximately 220 μm, although it can vary based on age and ocular diseases [14]. In our study, the subgroup with the greatest improvement in visual acuity (VA) was the one with a choroidal thickness between 210 and 279 μm, which was statistically significant. Three out of the four groups (70–139 μm, 210–279 μm, and 280–349 μm) demonstrated a significant mean reduction in VA, as measured by logMAR. However, the group with a choroidal thickness between 140 and 209 μm showed numerical improvement in VA, although not statistically significant, which may be attributed to the limited number of patients in each subgroup.

After treatment, the mean choroidal thickness showed a statistically significant reduction (p = 0.03) compared to baseline, consistent with a previous study by Kang et al. [13]. Patients with thicker choroids at the beginning of the study may have had a greater potential for recovery due to retained choriocapillaris. Interestingly, the group with the thickest choroids at baseline (280–349 μm) demonstrated the greatest reduction in choroidal thickness, although the result was not statistically significant. On the other hand, the group with the thinnest choroids (70–139 μm) was the only one to show a statistically significant mean reduction in choroidal thickness. Although the absolute value of reduction in thicker choroids is greater than in thinner choroids, when applying the statistical method, the reduction in thinner choroids was comparatively greater than in thicker choroids.

In our study, the group with the thinnest choroids (70–139 μm) exhibited the least positive response in terms of VA, with a statistically significant mean reduction of 0.35 in logMAR. However, the difference was relatively small compared to the groups with thicker choroids (210–279 μm and 280–349 μm), which showed respective mean reductions of 0.40 and 0.41 in logMAR, also with statistical significance. Notably, the group with choroidal thickness closest to the normal range (subgroup = 210–279 μm) demonstrated the most significant improvement in post-treatment VA, with a mean reduction of 0.40 and statistical significance (p-value = 0.011). This suggests that patients with choroidal thickness within or close to the normal range tend to experience more substantial improvements in VA.

The significant reduction in subfoveal choroidal thickness observed after intravitreal aflibercept injections in this study is consistent with previous findings in patients with neovascular AMD, polypoid choroidal vasculopathy, and angiomatous retinal proliferation [15–19]. The decrease in choroidal thickness may be associated with the vascular hyperpermeability of the choroid. Patients with thicker choroids may have a greater potential for recovery due to retained choriocapillaris.

The pachychoroid disease spectrum encompasses various conditions, including central serous chorioretinopathy, pachychoroid pigment epitheliopathy, focal choroidal excavation, peripapillary pachychoroid syndrome, pachychoroid neo vasculopathy, and pachychoroid neovascular type 1 macular neovascularization. Previous evidence suggests that patients with pachychoroid disease complicated by exudative neovascularization have a longer retreatment-free interval compared to those with neovascular AMD after an initial loading dose of anti-VEGF therapy. This difference has been attributed to lower VEGF secretion in pachychoroid disease compared to neovascular AMD. Patients with pachychoroid diseases and neovascular membranes have shown a more favorable response to anti-VEGF treatment, including aflibercept, with improved visual acuity and prognosis, albeit with a lower frequency of intravitreal injections [20–22].

Intravitreal aflibercept therapy can further reduce the hyperpermeability of retained choriocapillaris in these patients, leading to a significant decrease in subfoveal choroidal thickness [19]. Patients with thinner choroids may have fewer retained choriocapillaris, resulting in less substantial reductions in choroidal thickness after treatment. Therefore, it is less likely to achieve a significant decrease in choroidal vascular hyperpermeability in these patients, which may contribute to less significant changes in choroidal thickness. Furthermore, a thinner choroid in patients with AMD may indicate a more advanced and severe disease stage, potentially limiting the effectiveness of treatment. It is speculated that neovascular AMD with the thinnest choroids presents a more impaired choroidal circulation and a more prolonged, severe disease status, resulting in a diminished treatment response and poorer visual outcomes after intravitreal anti-VEGF injections [13, 23–25].

The average macular thickness of patients significantly decreased after the injections, as confirmed by statistical analysis (p = 0.001), consistent with several previous studies [10, 11, 23, 26].

We identified a statistically significant association between the difference in pre/post choroidal thickness and the pretreatment values of macular and choroidal thickness. Specifically, for every 1-micron increase in pretreatment macular thickness, the pre/post choroidal thickness difference value decreased by 0.31 microns. Similarly, for every 1-micron increase in pretreatment choroidal thickness, the pre/post choroidal thickness difference value decreased by 0.31 microns. Additionally, there was a statistically significant association between the difference in pre/post macular thickness and the pretreatment macular thickness value. For every 1-micron increase in pretreatment macular thickness, the pre/post macular thickness difference value decreased by 0.68 microns. However, no association was found between pretreatment choroidal thickness and pre/post macular thickness (p > 0.05). These comparisons suggest that greater reductions in choroidal thickness are associated with greater reductions in macular thickness, supporting the hypothesis that patients with greater subfoveal choroidal thickness have a better treatment response [23].

These correlations, not previously reported inliterature, could complement OCT-SS analysis in assessing treatment response and provide an additional objective parameter for analyzing the response to intravitreal aflibercept injections in patients with neovascular AMD.

The intraocular pressure (IOP) of patients before the injections was within the normal range and remained so after the applications, with only minor and physiologically irrelevant fluctuations. However, no other studies were found in literature to compare IOP in anti-VEGF treatment, including aflibercept, in neovascular AMD.

However, it is important to acknowledge the limitations of our study, including the small sample size and relatively short follow-up period. Further research with larger cohorts and long-term follow-up is necessary to validate and expand upon these findings.

## Conclusion

In conclusion, our study demonstrated significant improvements in visual acuity and reductions in subfoveal choroidal and macular thickness with OCT-SS following intravitreal aflibercept injections in patients with neovascular AMD. The correlation between subfoveal choroidal thickness and visual outcomes highlights the importance of choroidal thickness as a predictive factor for treatment response. Additionally, we observed correlations between pretreatment macular thickness and the changes in choroidal thickness, suggesting a potential new objective parameter for evaluating the response to anti-VEGF intravitreal injections in patients with neovascular AMD.

It is worth noting that our study is unique in literature, as it presents statistically significant correlations between pretreatment macular thickness, post-treatment choroidal thickness, and the variation in choroidal thickness using OCT-SS. These findings contribute to a better understanding of the relationship between choroidal and macular characteristics and treatment outcomes in neovascular AMD. Overall, our study provides valuable insights into the role of choroidal thickness in predicting treatment response and highlights the potential utility of assessing macular and choroidal characteristics as objective parameters in the management of neovascular AMD.

## Abbreviations

AMD: Age-related macular degeneration
CNV: Choroidal neovascularization
IOP: Intraocular pressure
OCT-SS: Optical coherence tomography swept source
OCT: Optical coherence tomography
RPE: Retinal pigmented epithelium
VA: Visual acuity;
VEGF: Anti-vascular endothelial growth factor
